# Antioxidant supplementation for the prevention of post-endoscopic retrograde cholangiopancreatography pancreatitis: a meta-analysis of randomized controlled trials

**DOI:** 10.1186/1475-2891-12-23

**Published:** 2013-02-11

**Authors:** Wan-Jie Gu, Chun-Yin Wei, Rui-Xing Yin

**Affiliations:** 1 Department of Anaesthesiology, The First Affiliated Hospital, Guangxi Medical University, Nanning, Guangxi, People’s Republic of China; 2 Department of Cardiology, Institute of Cardiovascular Diseases, The First Affiliated Hospital, Guangxi Medical University, 22 Shuangyong Road, Nanning, 530021, , Guangxi, People’s Republic of China

**Keywords:** Antioxidant, Endoscopic retrograde cholangiopancreatography, Pancreatitis

## Abstract

**Background:**

Acute pancreatitis remains the most common major complication of endoscopic retrograde cholangiopancreatography (ERCP). The pathogenesis of post-ERCP acute pancreatitis may be mediated by oxygen-derived free radicals, which could be ameliorated by antioxidants. Antioxidant supplementation may potentially prevent post-ERCP pancreatitis. We performed a meta-analysis of randomized controlled trials to evaluate the effect of prophylactic antioxidant supplementation compared with control on the prevention of post-ERCP pancreatitis.

**Methods:**

PubMed and Embase databases were searched to identify relevant trials. A standardized Excel file was used to extract data by two independent authors. Results were expressed as risk ratio (RR) with accompanying 95% confidence interval (CI). The meta-analysis was performed with the fixed-effects model or random-effects model according to heterogeneity.

**Results:**

Eleven studies involving 3,010 patients met our inclusion criteria. Antioxidant supplementation did not significantly decrease the incidence of post-ERCP pancreatitis (RR, 0.92; 95% CI, 0.65-1.32; *P* = 0.665). There was also no statistical difference in the severity grades between the antioxidant group and control group.

**Conclusions:**

Based on current evidence, antioxidant supplementation shows no beneficial effect on the incidence and the severity of post-ERCP pancreatitis; thus, there is currently a lack of evidence to support using antioxidants for the prevention of post-ERCP pancreatitis.

## Background

Acute pancreatitis is the most common major and severe complication of diagnostic and therapeutic ERCP, with the reported incidence ranging from 1.8% to 7.2% in large prospective series of nonselected patients [1-4]. The severity of post-ERCP pancreatitis can range from a mild course with one or two days prolonged hospitalization and full recovery to a devastating illness with hemorrhagic pancreatitis, pancreatic necrosis, multiorgan failure, and even death [5]. Because post-ERCP pancreatitis is predictable and possibly preventable, numerous attempts have been made to reduce the incidence and limit the severity of this complication. However, most of them have largely been disappointing.

Although the pathogenesis of post-ERCP pancreatitis is not clearly understood, a number of studies have demonstrated that an early step in the pathogenesis of acute pancreatitis is capillary endothelial injury manifested by an increase in capillary permeability [6,7]. Subsequent researches have suggested that this capillary injury may be mediated by oxygen-derived free radicals [8,9]. The manifestations of pancreatitis in experimental animal model can be ameliorated by blocking the action of oxygen-derived free radicals [10-12]. Based on the aforementioned findings, the idea of antioxidant supplementation for the prevention of post-ERCP pancreatitis seems rational and reasonable.

Two meta-analyses regarding allopurinol (an inhibitor of oxygen-derived free-radicals) on the prevention of post-ERCP pancreatitis have been published [13,14]. Both of them showed that allopurinol has no significant effect on the prevention of post-ERCP pancreatitis. However, one was only based on 4 trials and included relatively modest sample sizes [13], and another included half of the studies published in the abstract form, without access to the full data [14]. Moreover, the role of other antioxidants (such as N-acetylcysteine, β-carotene) has not been well established. Recently, several relevant randomized controlled trials (RCTs) regarding prophylactic antioxidant supplementation in preventing post-ERCP pancreatitis have been published. These reports were well-performed RCTs and included an additional more than 1,200 patients. We therefore undertook a meta-analysis of randomized controlled trials to evaluate the effect of prophylactic antioxidant supplementation compared with control on the incidence and the severity of post-ERCP pancreatitis.

## Methods

### Literature search and inclusion criteria

Literature searches of the PubMed and Embase databases (up to May 2012) were performed to identify RCTs that compared antioxidant versus control for the prevention of post-ERCP pancreatitis. The initial search terms were *antioxidant* and *pancreatitis*, filtered by *Humans* and*Randomized Controlled Trial*. In addition, the reference lists of identified studies were manually checked to include other potentially eligible trials. This process was performed iteratively until no additional articles could be identified.

The following inclusive selection criteria were applied: (i) study design: RCT; (ii) study population: adult patients undergoing ERCP; (iii) intervention: antioxidant supplementation (no matter what type and regimen applied); (iv) comparison intervention: placebo or no intervention; and (v) outcome measure: the incidence and the severity of post-ERCP pancreatitis.

### Data extraction and outcome measure

Two authors (WJG and CYW) independently extracted the following data from the selected studies: first author, year of publication, number of patients (antioxidant/control), patient characteristics, type of antioxidant, regimens of antioxidant supplementation (route, dosage, timing, frequency), study design, definition and severity of post-ERCP pancreatitis, and outcome data. Extracted data were entered into a standardized Excel file and were checked by another author (RXY). Any disagreements were resolved by discussion and consensus.

The outcome of interest was the incidence and the severity of post-ERCP pancreatitis. The definition of post-ERCP pancreatitis varied across studies, no standard definition was used in reported studies. In the majority of the studies, the definition of post-ERCP pancreatitis and the grading of its severity were based on the Cotton consensus criteria [15].

### Quality assessment

The methodological quality of each trial was evaluated using the Jadad scale [16]. The scale consists of three items describing randomization (0–2 points), blinding (0–2 points), and dropouts and withdrawals (0–1 points) in the report of a randomized controlled trial. A score of 1 is given for each of the points described. A further point is obtained where the method of randomization and/or blinding is given and is appropriate; whereas it is inappropriate a point is deducted. The quality scale ranges from 0 to 5 points. Higher scores indicate better reporting. The studies are said to be of low quality if the Jadad score is ≤ 2 and high quality if the score is ≥ 3 [17].

### Statistical analyses

All outcomes were expressed as RR with 95% CI. The Cochrane Q *x*^2^ test was used to detect heterogeneity of the effects, significant heterogeneity was defined as a *P* value of <0.05. A fixed-effects model or random-effects model was used, depending on the absence or presence of heterogeneity. *I*^2^ statistic was estimated to describe the percentage of the variability attributable to heterogeneity rather than sampling error. Studies with an *I*^2^ statistic of < 25% are considered to have no heterogeneity, those with an *I*^2^ statistic of 25% to 50% are considered to have low heterogeneity, those with an *I*^2^ statistic of 50% to 75% are considered to have moderate heterogeneity, and those with an *I*^2^ statistic of > 75% are considered to have high heterogeneity [18]. Whenever heterogeneity was present, sensitivity analyses based on sample size, study quality, and omitting one study in each turn were carried out to identify potential sources. We also investigated the influence of a single study on the overall pooled estimate by omitting one study in each turn.

Potential publication bias was assessed by visually inspecting of the Begg funnel plot in which the RRs were plotted against their SEs. The presence of publication bias was also evaluated by using the Begg and Egger tests [19,20]. A *P* value less than 0.05 was judged as statistically significant, except where otherwise specified. All statistical analyses were performed using STATA version 11.0 (Stata Corporation, College Station, Texas, USA).

## Results

### Study identification and selection

The PubMed and Embase search identified 42 and 25 potential studies, respectively. A total of 67 RCTs were identified by the initial database search. Thirteen RCTs were excluded because of duplicate studies and 43 RCTs were excluded based on the titles and abstracts (reviews, nonrandomized studies, or not relevant to our analysis). The remaining 11 were then retrieved for full text review. Finally, eleven RCTs met inclusion criteria and were included in the analysis [21-31].

### Study characteristics

The main characteristics of eleven RCTs included in this meta-analysis are presented in Table 1 and the definition and severity of post-ERCP pancreatitis of each included trial are described in Table 2. These studies were published between 1999 and 2011. The size of the RCT ranged from 40 to 701 (total 3,010). Among the 11 studies included here, all reported post-ERCP pancreatitis events [21-31], 8 reported mild and moderate post-ERCP pancreatitis events [22-26,28,29,31], and 6 reported severe post-ERCP pancreatitis events [22-24,26,28,29]. The median Jadad score of the studies included was 3 (range from 2 to 5).

**Table 1:** Main characteristics of randomized controlled trials included in the meta-analysis

**Study**	**No. of patients (Antioxidant/Control)**	**Patient characteristics**	**Antioxidant supplement**	**Intervention**		**Study design**	**Jadad score**
				**Antioxidant**	**Control**		
Wollschläger et al. [21]	40 (20/20)	Adult patients undergoing ERCP	Selenite	Selenite, intravenously, 1 mg bolus/2 x 1 mg infusion, l d before ERCP	Control, no prophylaxis	Randomized, controlled	2
Budzyńska et al. [22]	200 (99/101)	Adult consecutive patients undergoing elective ERCP	Allopurinol	Allopurinol, orally, 200 mg, 15 h and 3 h before ERCP	Placebo, orally, 200 mg, 15 h and 3 h before ERCP	Randomized, placebo-controlled	3
Lavy et al. [23]	321 (141/180)	Adult consecutive patients undergoing ERCP	β-carotene	β-carotene, orally, 2 g, 12 h before ERCP	Placebo, orally, 2 g, 12 h before ERCP	Randomized, double-blind, placebo-controlled	5
Katsinelos et al. [24]	249 (124/125)	Adult consecutive patients undergoing diagnostic or therapeutic ERCP	NAC	NAC, intravenously, 70 mg/kg 2 h before, and 35 mg/kg at 4 h intervals for 24 h after ERCP	Placebo (0.9% saline solution), intravenously, 70 mg/kg 2 h before, and 35 mg/kg at 4 h intervals for 24 h after ERCP	Randomized, double-blind, placebo-controlled	5
Katsinelos et al. [25]	243 (125/118)	Adult consecutive patients undergoing diagnostic or therapeutic ERCP	Allopurinol	Allopurinol, orally, 600 mg, 15 h and 3 h before ERCP	Placebo, orally, 600 mg, 15 h and 3 h before ERCP	Randomized, double-blind, placebo-controlled	5
Mosler et al. [26]	701 (355/346)	Adult patients undergoing diagnostic or therapeutic ERCP	Allopurinol	Allopurinol, orally, 4 h (600 mg) and 1 h (300 mg) before ERCP	Placebo (similar pill without the active drug), orally, 4 h (600 mg) and 1 h (300 mg) before ERCP	Randomized, double-blind, placebo-controlled	5
Milewski et al. [27]	106 (55/51)	Adult consecutive patients undergoing ERCP	NAC	NAC, two 600 mg given orally 24 h and 12 h before ERCP and 600 mg given intravenously for 2 d after ERCP	Placebo (isotonic saline), intravenously, twice a day for 2 d after ERCP	Randomized, placebo-controlled	2
Kapetanos et al. [28]	320 (158/162)	Adult patients undergoing ERCP	Pentoxifylline	Pentoxifylline, orally, 400 mg, beginning the day before ERCP (2 and 10 PM) until the night after ERCP (6 AM and 2 and 10PM)	No intervention	Randomized, controlled	3
Romagnuolo et al. [29]	586 (293/293)	Adult patients undergoing ERCP	Allopurinol	Allopurinol, orally, 300 mg, 1 h before ERCP	Placebo, orally, 300 mg, 1 h before ERCP	Randomized, double-blind, placebo-controlled	5
Martinez et al. [30]	170 (85/85)	Adult patients undergoing ERCP	Allopurinol	Allopurinol, orally, 300 mg, 15 h and 3 h before ERCP	Placebo, orally, 300 mg, 15 h and 3 h before ERCP	Randomized, placebo-controlled	3
Abbasinazari et al. [31]	74 (29/45)	Adult patients undergoing ERCP	Allopurinol	Allopurinol, orally, 300 mg, 15 h and 3 h before ERCP	Placebo, orally, 300 mg, 15 h and 3 h before ERCP	Randomized, double-blind, placebo-controlled	3

ERCP = endoscopic retrograde cholangiopancreatography, NAC = N-acetylcysteine.

**Table 2:** Definition and severity of post-ERCP pancreatitis

**Study**	**Definition of post-ERCP pancreatitis**	**Severity of post-ERCP pancreatitis**
Wollschläger et al. [21]	Presence of abdominal pain attributed to pancreatitis, in association with a serum lipase or amylase level greater than 2 times the upper limit of normal	NA
Budzyńska et al. [22]	Presence of abdominal pain attributed to pancreatitis, together with a need for an unplanned hospitalization or an extension of a planned hospitalization by at least 2 d, and a serum amylase at least 3 times above the upper limit of normal at 24 hours after ERCP	Mild: symptoms lasting up to 3 d and pancreas normal on the CT scan; moderate: requiring specific therapeutic measures for 4–10 d, Balthazar’s grade B/C on CT; severe: local or systemic complications for more than 10 d, Balthazar’s grade D/E on CT, or death
Lavy et al. [23]	Presence of abdominal pain attributed to pancreatitis, in association with elevated amylase levels at least 3 times higher than the upper limit of normal	Mild: requiring 2–3 d of hospitalization; moderate: requiring 4–10 d of hospitalization; severe: requiring 10 d of hospitalization or requiring surgical intervention or leading to death
Katsinelos et al. [24]	Presence of abdominal pain attributed to pancreatitis, together with a need for an unplanned hospitalization or an extension of a planned hospitalization by at least 2 d, and a serum amylase at least 3 times above the upper limit of normal at 24 hours after ERCP	Mild: symptoms persisting for 3 d and a normal appearance of the pancreas by US and/or CT; moderate: requirement for specific therapeutic measures for 4 to 10 d (Balthazar’s grade B/C on CT); severe: local or systemic complications for more than 10 d after ERCP (Balthazar’s grade D/E) or death
Katsinelos et al. [25]	Presence of abdominal pain attributed to pancreatitis, together with a need for an unplanned hospitalization or an extension of a planned hospitalization by at least 2 d, and a serum amylase at least 3 times above the upper limit of normal at 24 hours after ERCP	Mild: symptoms persisting for 3 d and a normal appearance of the pancreas by US and/or CT; moderate: requirement for specific therapeutic measures for 4 to 10 d (Balthazar’s grade B/C on CT); severe: local or systemic complications for more than 10 d after ERCP (Balthazar’s grade D/E) or death
Mosler et al. [26]	New-onset or increased abdominal pain lasted for more than 24 h, caused an unplanned admission of an outpatient for more than one night, or prolonged a planned admission of an inpatient, and was associated with a serum amylase level increase of at least 3 times above normal, at approximately 18 hours (the next morning) after ERCP	Mild: hospitalization lasted 2–3 d; moderate: hospitalization lasted 4–10 d; severe: hospitalization was prolonged for more than 1 0 d or any of the following occurred: hemorrhagic pancreatitis, pancreatic necrosis, pancreatic pseudocyst, or the need for percutaneous drainage or surgery
Milewski et al. [27]	Clinical features consistent with acute pancreatitis beginning after ERCP and lasting for at least 24 h, associated with increase in serum amylase levels greater than 5 times above normal	NA
Kapetanos et al. [28]	Presence of abdominal pain attributed to pancreatitis, together with a need for an unplanned hospitalization or an extension of a planned hospitalization by at least 2 d, and a serum amylase at least 3 times above the upper limit of normal at 24 hours after ERCP	Mild: clinical pancreatitis and serum amylase at least 3 times higher than normal at more than 24 h after ERCP, requiring admission or prolongation of planned admission for 2–3 d; moderate: required hospitalization for 4–10 d; severe: required hospitalization for more than 10 d, an intervention (percutaneous drainage or surgery), or if a pseudocyst was diagnosed
Romagnuolo et al. [29]	Presence of typical pancreatic pain (epigastric pain often radiating into the back and associated with nausea and/or vomiting) requiring medical attention, in association with a serum lipase or amylase level greater than 2 times the upper limit of normal	NA
Martinez et al. [30]	Serum amylase was above 600 UI/L or 3 times above the normal value and the patient had a sharp pain irradiating to the back and nausea or vomiting	Ranson’s criteria (Parameter: At admission: age >55 y, WBC count >16,000/uL, serum glucose level >11.1 mmol/L, SLDH/ALT >350 IU/L, AST level >250 IU/L; During initial 48 h: hematocrit: decrease of more than 0.10, BUN level: increase of more than 5 mg/dL, calcium: <2 mmol/L, PaO_2_<60 mm Hg, base deficit >4 mmol/L, fluid sequestration >6L): mild: two or fewer grave signs; severe: more than six grave signs.
Abbasinazari et al. [31]	NA	Mild: amylase concentration at least 3 times above upper limit of normal at more than 24 h after ERCP requiring admission for 2–3 d; moderate: admission for 4–10 d; severe: admission for more than 10 d

ERCP = endoscopic retrograde cholangiopancreatography, NA = not available, AST = asparate aminotransferase, BUN = blood urea nitrogen, SLDH/ALT = serum lactate dehydrogenate to alanine aminotransferase ratio, WBC = white blood cell.

All these patients were older than 18 years and scheduled for ERCP. The selected trials used different types of antioxidant, including sodium selenite [21], allopurinol [22,25,26,29-31], N-acetylcysteine [24,27], β-carotene [23], and pentoxifylline [28]. These antioxidants were administered orally or intravenously by different regimens and formulations. Two studies used an intravenous route to administer antioxidant [21,24], and the remaining nine studies orally applied antioxidant during the perioperative period [22,23,25-31]. Dosage, timing, and frequency of these antioxidants are various.

### The incidence of post-ERCP pancreatitis

**Figure 1:**
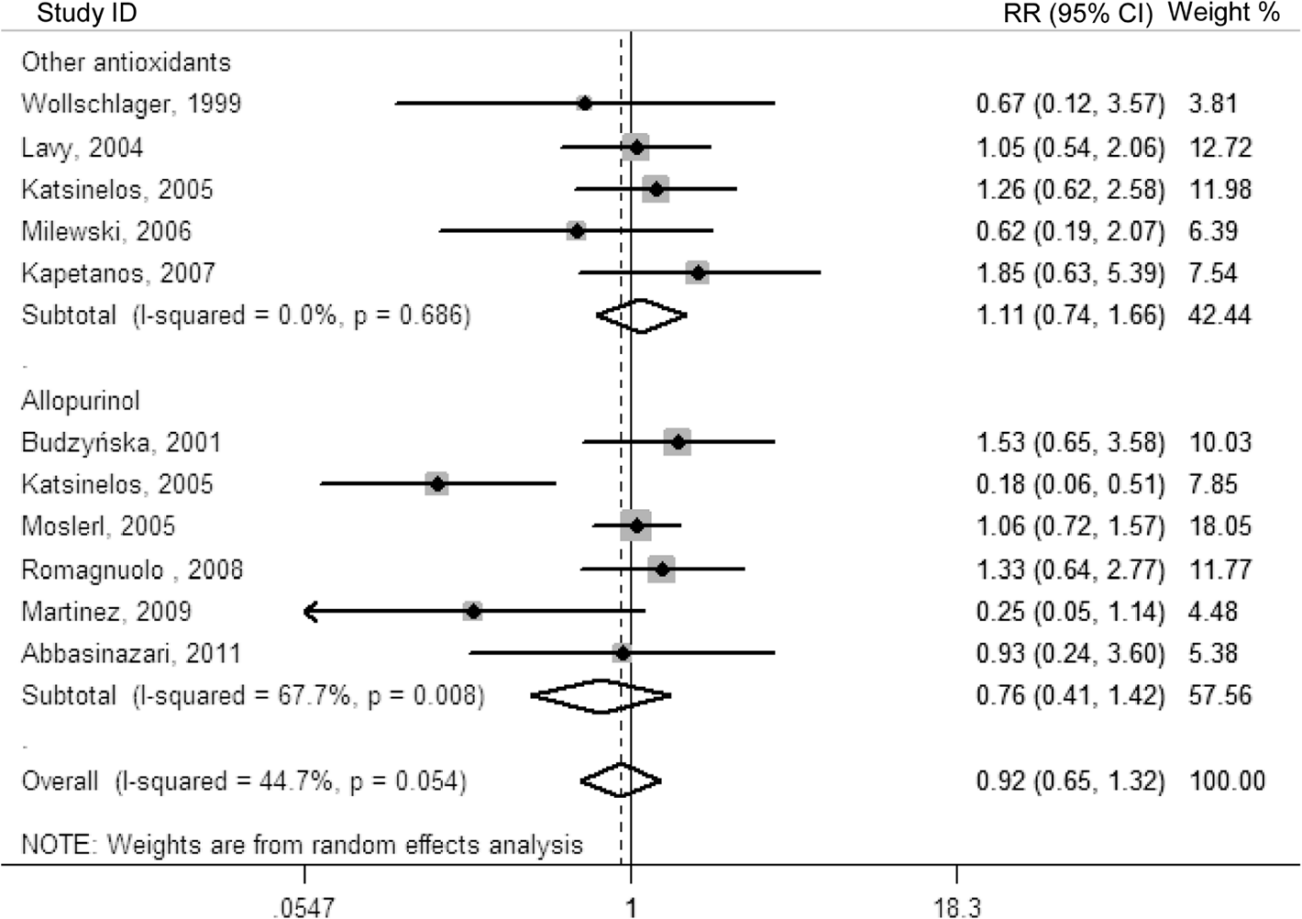
Forest plot showing the effect of antioxidant supplementation on the incidence of post-ERCP pancreatitis.

The outcome data of each included trial are described in Table 3. A total of 3,010 patients were included in the ten trials comparing antioxidant with control for the prevention of post-ERCP pancreatitis (1,484 in the antioxidant group and 1,526 in the control group). Altogether, 266 patients developed post-ERCP pancreatitis, 127 in the antioxidant group and 139 in the control group. Antioxidant supplementation were not associated with a significant reduction in the incidence of post-ERCP pancreatitis (RR, 0.92; 95% CI, 0.65-1.32; *P* = 0.665), with significant heterogeneity among the studies (*I*^2^ = 44.7%; *P* = 0.054). Furthermore, when trials were divided by the type of antioxidant, there was also no significant decrease in post-ERCP pancreatitis (RR for trials with allopurinol: 0.76; 95% CI: 0.41-1.42; *P* = 0.396; and RR for trials with other antioxidants: 1.11; 95% CI: 0.74-1.66; *P* = 0.622) (Figure 1).

**Table 3:** Outcome data of randomized controlled trials included in the meta-analysis

**Study**	**Antioxidant group**					**Control group**				
	**No. of patients (n)**	**No. of PEP (n)**	**PEP stratified by severity**			**No. of patients (n)**	**No. of PEP (n)**	**PEP stratified by severity**		
			**Mild**	**Moderate**	**Severe**			**Mild**	**Moderate**	**Severe**
Wollschläger et al. [21]	20	2	NA	NA	NA	20	3	NA	NA	NA
Budzyńska et al. [22]	99	12	9	2	1	101	8	5	3	0
Lavy et al. [23]	141	14	10	4	0	180	17	9	4	4
Katsinelos et al. [24]	124	15	8	7	0	125	12	7	5	0
Katsinelos et al. [25]	125	4	4	0	0	118	21	8	11	2
Mosler et al. [26]	355	46	28	16	2	346	42	24	16	2
Milewski et al. [27]	55	4	NA	NA	NA	51	6	NA	NA	NA
Kapetanos et al. [28]	158	9	6	1	2	162	5	4	0	1
Romagnuolo et al. [29]	293	16	8	6	2	293	12	4	6	2
Martinez et al. [30]	85	2	NA	NA	NA	85	8	NA	NA	NA
Abbasinazari et al. [31]	29	3	2	1	0	45	5	3	2	0

ERCP = endoscopic retrograde cholangiopancreatography, PEP = post-ERCP pancreatitis, NA = not available.

Subsequently, we performed sensitivity analyses to explore potential source of heterogeneity. Exclusion of two trials which had a modest sample size (N ≤ 100) yielded similar results (RR 0.92; 95% CI 0.62-1.39; *P* = 0.702), with moderate heterogeneity (*I*^2^ = 55.2%; *P* = 0.022) [21,31]. Exclusion of two studies with low quality (Jadad score ≤ 2) did not change the pooled results substantially (RR 0.96; 95% CI 0.64-1.43; *P* = 0.823), yet heterogeneity was still present (*I*^2^ = 53.6%; *P* = 0.028) [21,27]. Exclusion of one study conducted by Katsinelos et al. changed the overall estimate little (RR, 1.08; 95% CI, 0.85–1.37; *P* = 0.531), but no evidence of heterogeneity was observed among the remaining studies (*I*^2^ = 0%; *P* = 0.651) [25]. Further exclusion of any single study also did not materially alter the overall combined RR (data not shown).

### The severity of post-ERCP pancreatitis

**Figure 2:**
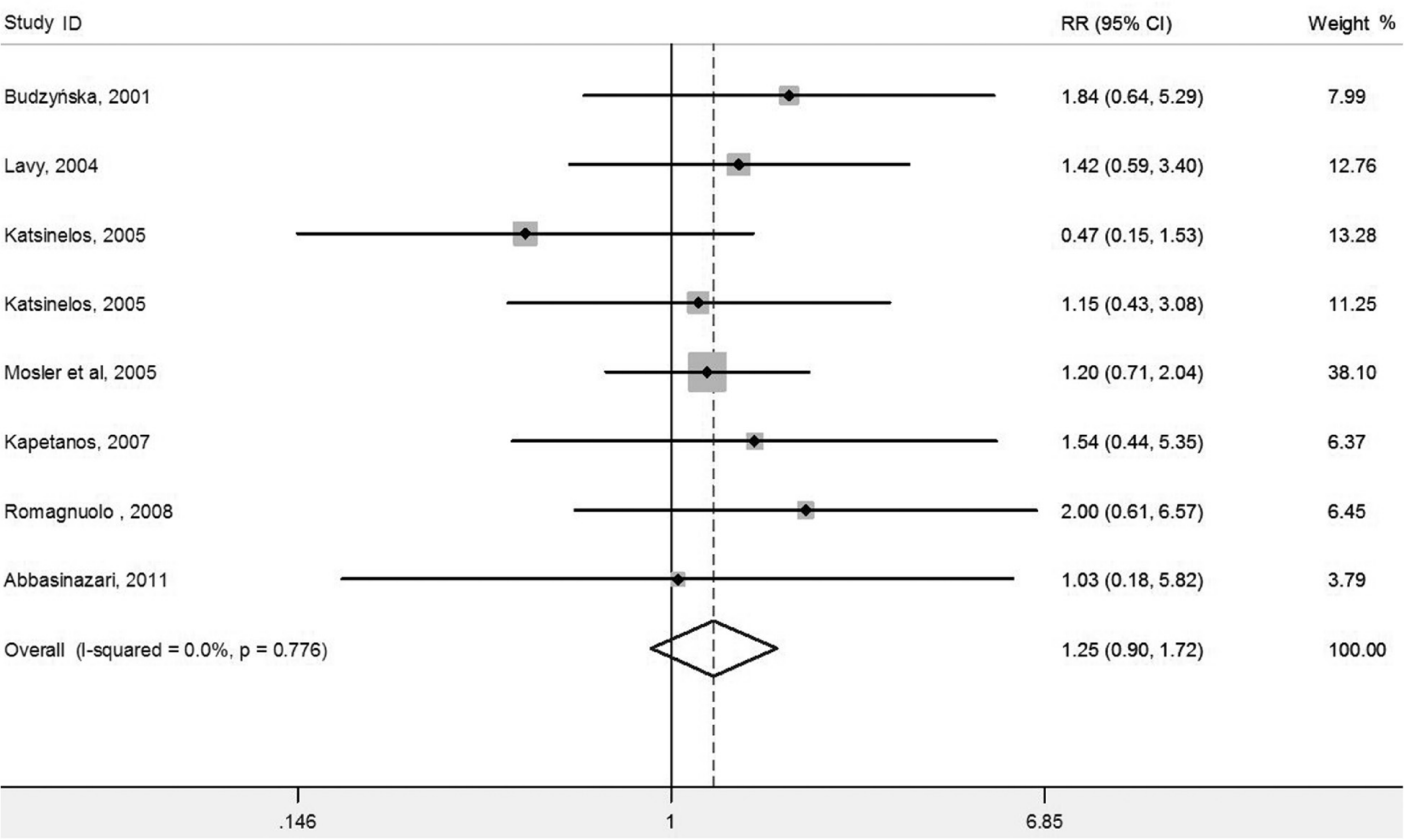
Forest plot showing the effect of antioxidant supplementation on the incidence of mild post-ERCP pancreatitis.

**Figure 3:**
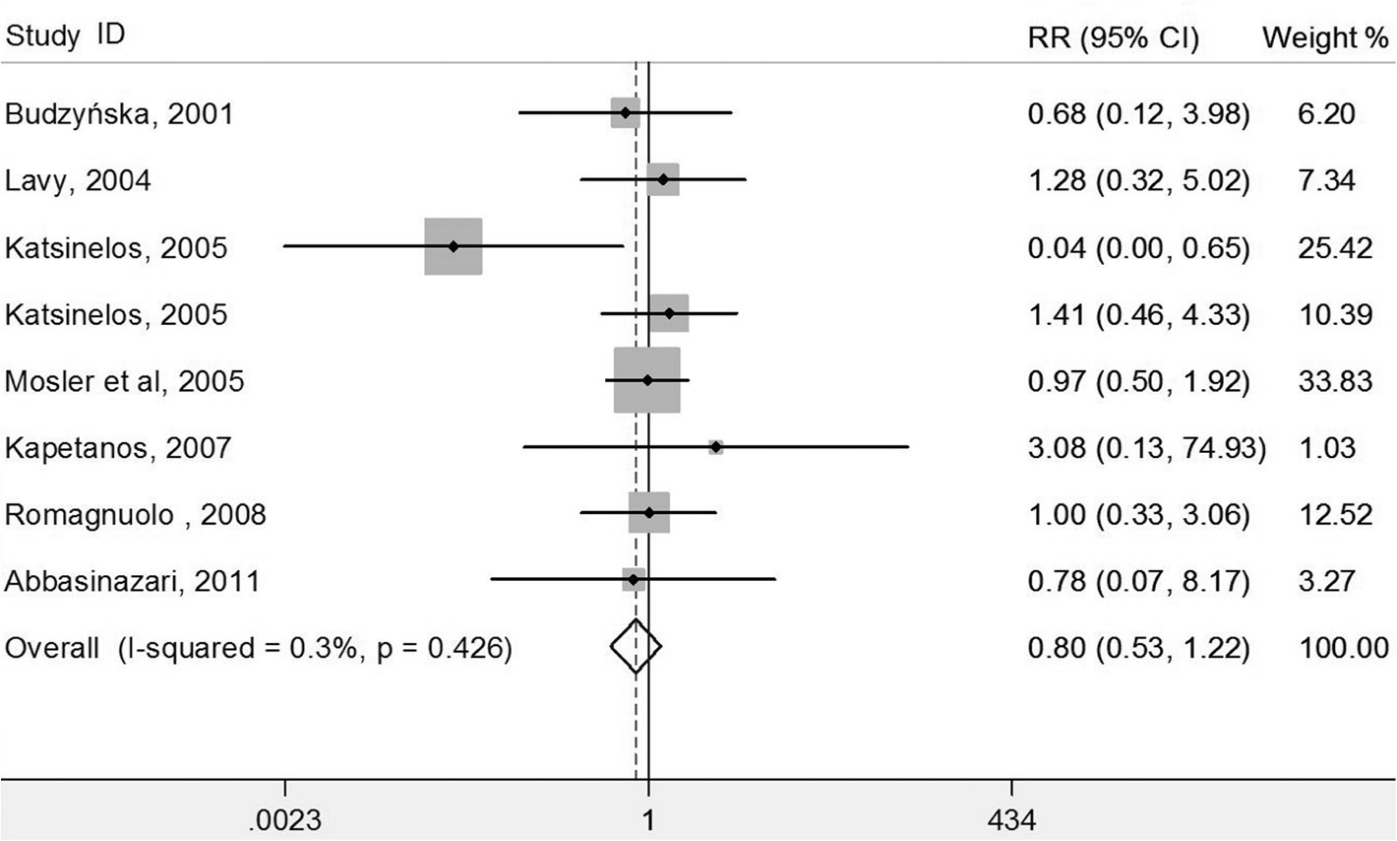
Forest plot showing the effect of antioxidant supplementation on the incidence of moderate post-ERCP pancreatitis.

**Figure 4:**
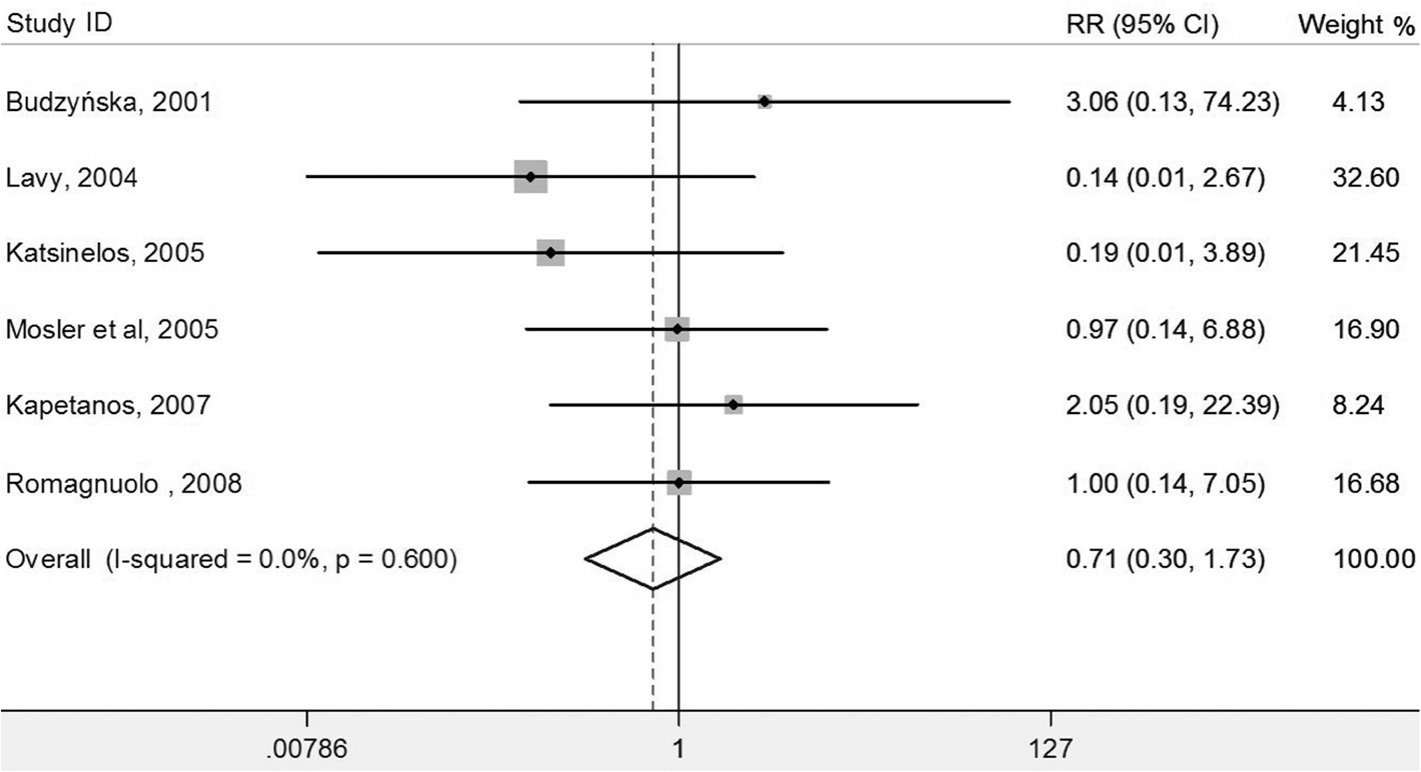
Forest plot showing the effect of antioxidant supplementation on the incidence of severe post-ERCP pancreatitis.

We also performed meta-analyses according to the grade to explore the effect of antioxidant on the severity of post-ERCP pancreatitis. Antioxidant supplementation had no impact on mild post-ERCP pancreatitis (eight RCTs, RR 1.25, 95% CI 0.90-1.72; *P* = 0.183; *I*^2^ = 0%; heterogeneity *P* = 0.776) (Figure 2), moderate post-ERCP pancreatitis (eight RCTs, RR 0.80, 95% CI 0.53-1.22; *P* = 0.304; *I*^2^ = 0.3%; heterogeneity *P* = 0.426) (Figure 3), or severe post-ERCP pancreatitis (six RCTs, RR 0.71, 95% CI 0.30-1.73; *P* = 0.455; *I*^2^ = 0%; heterogeneity *P* = 0.600) (Figure 4).

### Publication bias

**Figure 5:**
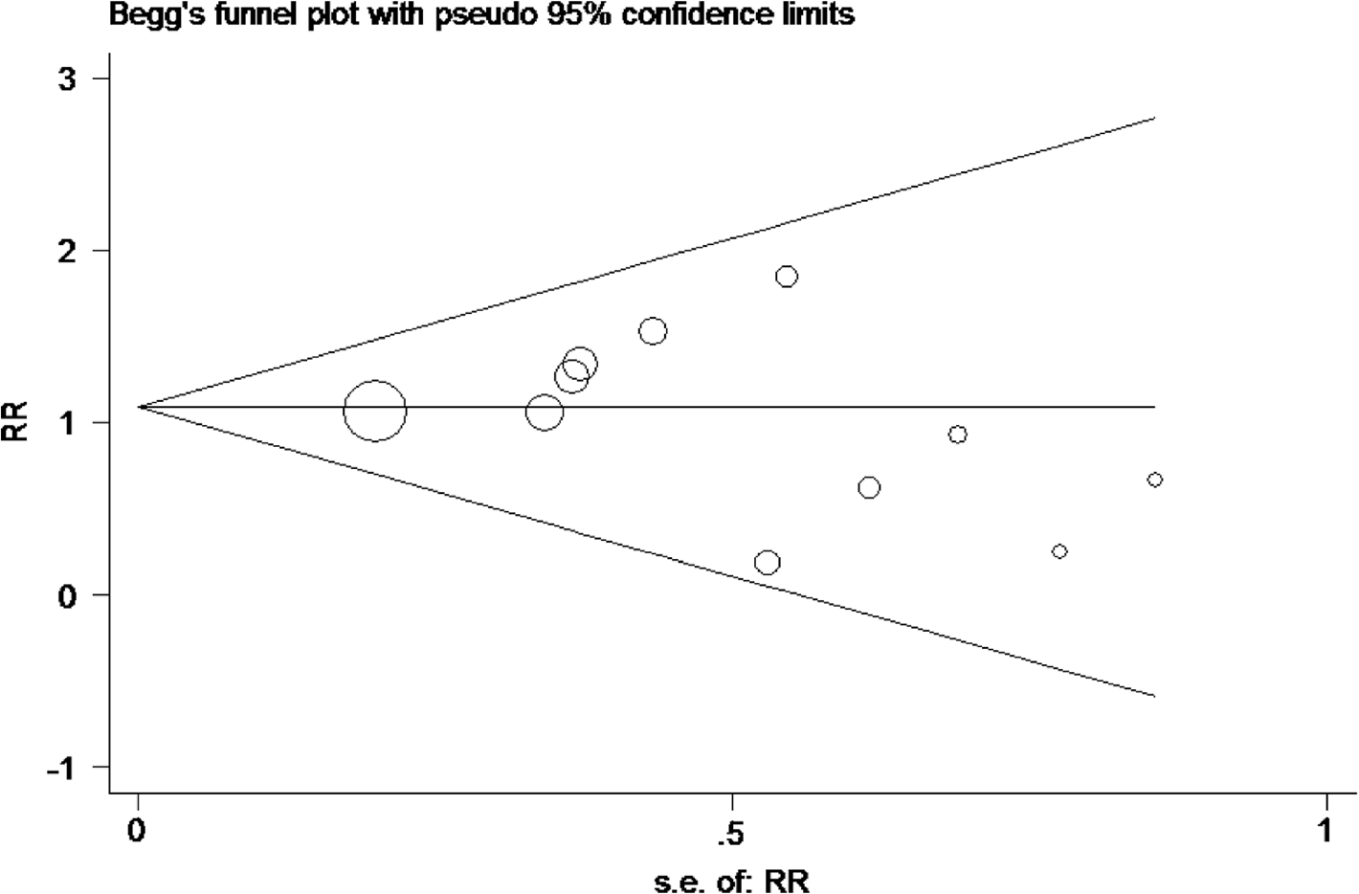
Tests for publication bias for RR of the incidence the incidence of post-ERCP pancreatitis.

Assessment of publication bias using Egger’s and Begg’s tests showed that there was no potential publication bias among the included trials (Egger’s test, *P* = 0. 443; Begg’s test, *P* = 0.533, Figure 5).

## Discussion

This is a further meta-analysis to evaluate the effect of prophylactic antioxidant supplementation on the prevention of post-ERCP pancreatitis. The present meta-analysis of ten randomized controlled trials showed that antioxidant supplementation did not significantly decrease the incidence of post-ERCP pancreatitis. In addition, antioxidant supplementation also showed no beneficial effect on the severity of post-ERCP pancreatitis.

There have been two published meta-analyses of allopurinol (an inhibitor of oxygen-derived free-radicals) for post-ERCP pancreatitis prevention [13,14]. Both of them showed that the use of allopurinol was not associated with reduction in the incidence of post-ERCP pancreatitis. Our meta-analysis expands on these two earlier meta-analyses to provide a better characterization of the evidence base for antioxidant supplementation in preventing post-ERCP pancreatitis. First, in our analysis, there are more enlarged sample sizes than the previous analysis, giving greater power to evaluate this effect. Second, we were more capable of evaluating the effects of other antioxidants (such as N-acetylcysteine, β-carotene, and selenite) on post-ERCP pancreatitis prevention. Furthermore, we also were able to evaluate the effect of antioxidant supplementation on the severity of post-ERCP pancreatitis.

Based on the previous meta-analysis, we furthermore included other seven recent RCTs [21,23,25,27,28,30,31]. With the added statistical power of having 2,970 cases, the present meta-analysis suggested that antioxidant supplementation did not significantly decrease the incidence of post-ERCP pancreatitis, which was in line with the previous meta-analysis. Moreover, exclusion of any single study and sensitivity analyses based on various exclusion criteria did not materially alter the pooled results, which adds robustness to our main finding. We also assessed the effect of antioxidant supplementation on the severity of post-ERCP pancreatitis, but failed to find significant alteration.

There was significant heterogeneity between studies in the overall analysis, which was not surprising given the differences in characteristics of populations, antioxidant supplementation, and definitions of pancreatitis. Our sensitivity analyses suggest that one trial conducted by Katsinelos et al. probably contributed to the heterogeneity [25]. For this study, only 43 patients in the allopurinol group underwent biliary sphincterotomy vs. 87 in the placebo group; thus, there was significant difference in the proportions of patients with biliary sphincterotomy between the two groups (*P* < 0.001). Besides, exclusion of the trial in our meta-analysis would not change our result; antioxidant supplementation still did not significantly decrease the incidence of post-ERCP pancreatitis (2,767 patients; fixed-effects model: RR, 1.08; 95% CI, 0.85–1.37; *P* = 0.531; data from nine trials) [21-23,25-31].

Furthermore, antioxidant therapy failed to prevent the onset of post-ERCP pancreatitis in almost all trials. Only one clinical trial in which 600 mg of allopurinol was administered twice before ERCP showed a significant decrease in the rate of post-ERCP pancreatitis. The positive result may be attributed to the high doses of allopurinol. Wisner and Renner found that only high doses of allopurinol were effective in preventing pancreatic edema and an increase in serum amylase in caerulein-induced pancreatitis in rats [11]. Low doses of allopurinol were not effective in fully suppressing xanthine oxidase, causing a “leakage” of excessive radicals.

Most of the included RCTs did not reported adverse effects of antioxidant during the study period. We found that only 1 report indicated different adverse effects of N-acetylcysteine. In the report [25], side effects most commonly attributable to N-acetylcysteine (i.e., skin rash, nausea, vomiting, and diarrhea) were observed with increased frequency in the N-acetylcysteine group (25% vs. 3.2%, N-acetylcysteine and placebo groups, respectively; *P* < 0.001).

The results of this meta-analysis must be interpreted cautiously in light of the strengths and limitations of the included trials. A major strength of this study is that all the included original studies used a randomized controlled design and most of them were well-performed, high-quality (Jadad score ≥ 3 in nine trials). In addition, with the enlarged sample size, we have enhanced statistical power to provide more precise and reliable effect estimates. Although the present analysis represents a complete summary of the current available evidence for antioxidant supplementation in preventing post-ERCP pancreatitis, it also serves to highlight limitations that remain. One potential limitation is the various types of antioxidant used and the lack of standard regimens of antioxidant applied in the randomized trials to date. These factors may result in the heterogeneity and have potential impact on our results. Furthermore, the variety of criteria was used to define pancreatitis. In the majority of the studies, the definition of post-ERCP pancreatitis and the grading of its severity were based on the Cotton consensus criteria [15]. Finally, exploration of the impact of antioxidant supplementation on other clinically meaningful endpoints including hyperamylasemia and length of the hospital stay has not been sufficient because of sparse and inconsistent reporting across the reviewed studies.

## Conclusions

In conclusion, the results suggest that antioxidant supplementation shows no beneficial effect on the incidence and the severity of post-ERCP pancreatitis. There is currently a lack of evidence to support using antioxidants for the prevention of post-ERCP pancreatitis.

## Abbreviations

CI: Confidence interval; ERCP: Endoscopic retrograde cholangiopancreatography; RCT: Randomized controlled trial; RR: Relative risk.

## Competing interests

The authors declare that they have no competing interests.

## Authors’ contributions

WJG conceived the study, participated in the design, collected the data, and drafted the manuscript. CYW collected the data, and performed statistical analyses. RXY conceived the study, participated in the design, and helped to draft the manuscript. All authors read and approved the final manuscript.
